# A case of unusual presentation of Takayasu's arteritis

**DOI:** 10.4103/0301-4738.60090

**Published:** 2010

**Authors:** Debabrata Das, Kanchan Kumar Mondal, Biswarup Ray, Asim Chakrabarti

**Affiliations:** Department of Ophthalmology, R.G. Kar Medical College and Hospital, Kolkata, India

**Keywords:** Aorto-arteritis, branch retinal artery occlusion, Takayasu's arteritis

## Abstract

Takayasu's arteritis is a chronic inflammatory disease of the large and medium-sized arteries. It commonly involves the aorta with its branches and the pulmonary arteries. The retinal hemodynamics suggest that the carotid artery involvement causes diminished retinal blood flow. This is the pathogenetic mechanism of Takayasu's retinopathy with characteristic features of microaneurysms, arterio-venous anastomosis and non-perfused retinal areas. Our case presented as branch retinal artery occlusion with collaterals and iris neovascularization. The branch retinal artery, a small retinal artery occlusion in our case is an unusual presenting feature of Takayasu's aorto-arteritis.

In Takayasu's arteritis (TA) inflammation around the vasa vasorum of the large and medium-sized arteries contributes to the development of the clinicopathological manifestations.[1] Ophthalmologic manifestations in the disease commonly result from the ocular hypotension secondary to occlusion of the large and medium-sized arteries.[1–3] Branch retinal artery occlusion is rare in Takayasu's arteritis.[1] Here we present a case with branch retinal artery occlusion with iris neovascularization.

## Case Report

**Figure 1:**
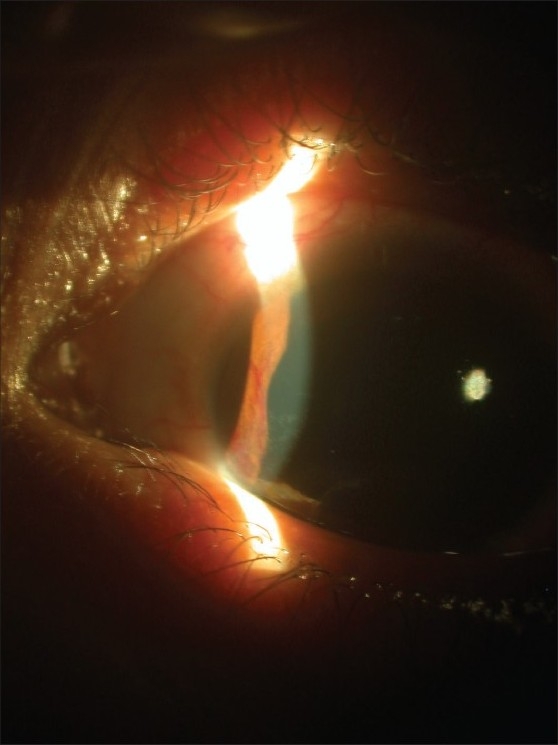
Slit-lamp photograph of the left eye showing iris neovascularization

**Figure 2:**
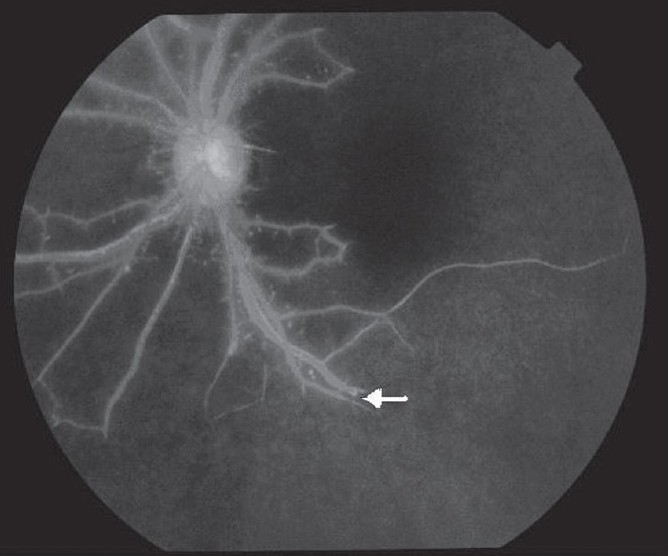
Fluorescein angiogram of the left eye showing non-filling of the tertiary branches of the lower temporal retinal vessel (white arrow) with collaterals and few hyperfluorescence points due to leakage of the dye

**Figure 3:**
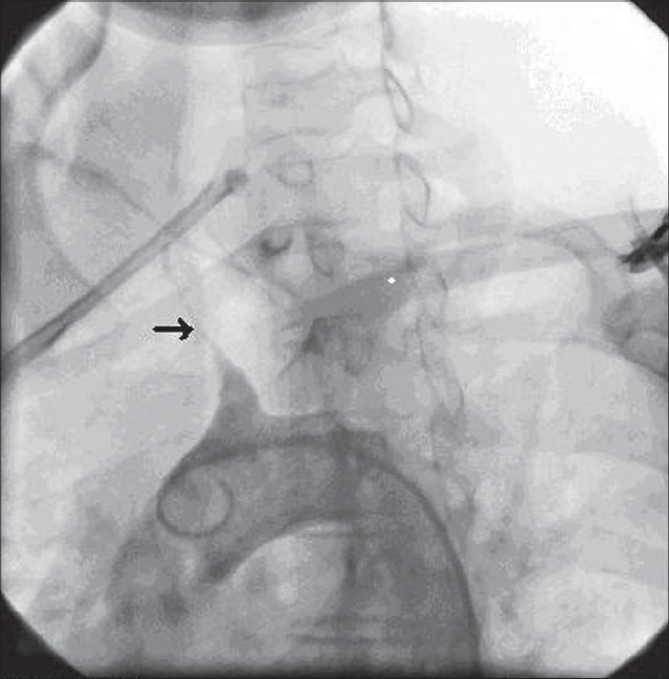
Angiography of the aortic arch showing Type I arteritis with complete occlusion of the left carotid, left subclavian artery and the right brachiocephalic artery (black arrow) extensively stenosed

A 16-year-old girl presented with dimness of vision in left eye and left-sided headache for four months. Her headache usually lasted for one to two hours and was neither preceded by aura nor associated with vomiting. There was no history of any other systemic and gynecological illness. Ocular examination revealed best-corrected visual acuity had been restricted to perception of light with accurate projection of rays in the left eye (LE) and 20/40 in the right eye (RE). Slit-lamp examination showed iris neovascularization in the LE [Fig. 1] and normal RE. Intraocular pressure (IOP) by applanation tonometry was 16 mm of Hg in each eye. Gonioscopy of both eyes revealed open angles without any neovascularization. Ophthalmoscopy revealed occlusion of the tertiary branches of the lower temporal branch retinal artery in LE and few microaneurysms. There was no arterio-venous anastomosis. Fundus fluorescein angiogram (FFA) showed delayed initial transit time in both eyes, non-filling of the tertiary branches of the lower temporal retinal vessel with collaterals formation and few hyperfluorescence points due to leakage of the dye [Fig. 2]. A detailed systemic examination was done as our patient had features of ocular ischemia. Systemic examination revealed absent arterial pulses in the left arm. The arterial pulses in the right arm were feeble. The blood pressure was unrecordable in the left upper limb with 80/60 mm of Hg in the right arm and 120/80 mm of Hg in the lower limbs. On routine hematological examination erythrocyte sedimentation rate (ESR) was 28 in the first hour by Westergren method. The biochemical test for serum urea and creatinine were normal. Serological test for Venereal Disease Research Laboratory(VDRL), anti- steptolysin O (ASO) and anti-nuclear antibody (ANA) titers were normal. A chest radiograph revealed no abnormality. Angiography of the aortic arch showed non-visualization of all branches of aorta except the right brachiocephalic branch that showed extensive stenosis in the third part [Fig. 3]. Renal angiogram was normal. Echodoppler study was normal. She was diagnosed as a case of aortic arch syndrome which is common in Takayasu's aorto-arteritis.

The patient was treated with oral prednisolone tablet 40 mg daily for three months. During the course of oral steroid therapy, slit-lamp biomicroscopy revealed no change in iris neovascularization. Gonioscopy of the LE showed open angle in all quadrants with minimal angle neovascularization and RE gonioscopy findings were normal. IOPs were recorded 16 mm Hg in the RE, 20 mm Hg in the LE by applanation tonometry. In her subsequent follow-up visits, the IOP increased to 28 mm Hg in the LE and gonioscopically we detected upper nasal quadrant synechial closure of the angle. We started antiglaucoma medication with topical Timolol maleate 0.5% in the LE and planned for panretinal photocoagulation in the same eye. However, the patient was lost to follow-up after five months.

Her vision improved to 20/120 in the involved eye after two months of oral steroid therapy. The blood pressure in the lower limbs was unchanged but had increased slightly in the arms following three months' treatment with oral steroids.

## Discussion

Takayasu's arteritis was first reported by M. Takayasu, a Japanese ophthalmologist in the year 1908.[3,4] TA commonly affects young adults in the second and third decade of life with female:male ratio 1.3:1 in India.[4] It is a chronic granulomatous necrotizing vasculitis predominantly affecting the aorta with its branches, pulmonary and coronary arteries.[3,4] TA is classified by angiographic morphology as Type I involving the aortic arch and its branches, Type II involving thoracoabdominal aorta and its branches, Type III involving both Type I and Type II, Type IV involving pulmonary artery with any of the other types.[4] The inflammation in TA causes varying degree of stenosis, occlusion or dilatation of the involved vessels.[3–7] The exact pathogenesis of the arteritis is still unknown.[1–8] Though tuberculosis, *streptococcal* infections, rheumatoid arthritis and other collagen vascular diseases had been debated for its etiology in the past, recently more emphasis has been given on immunopathological cause.[4] Recent studies strongly suggest association of interleukin-6 and RANTES (regulated on activation, normal T cell expressed and secreted) in the pathogenesis of the disease process.[8] Histopathology of the involved vessels suggests that the initial site of inflammation is around the vasa vasorum which are small arteries in the media and adventitia.[1,4] Similar primary small vessel involvement in other parts of the body including retinal vessels are the possible explanation of the clinico-pathological manifestations of TA.[1,5–7] Classical ophthalmological features are due to hypotension or hypertensive retinopathy.[3,4] The ocular features in the disease commonly result from the ocular hypotension secondary to occlusion of the arteries. The variable ocular presentations of TA depend on the parts of the carotid artery occluded, duration, rate of vascular insufficiency and development of collateral blood supply.[1] In 1976 Uyama and Asayama described typical features of Takayasu's retinopathy with dilatation of small vessels, capillary microaneurysm, arterio-venous anastomosis.[1,4] Hypertensive retinopathy secondary to renal artery occlusion may cause additional ocular manifestations.[1,4] The systemic small artery involvement has been described frequently in various publications of TA.[5–7] but documentation of the retinal artery involvement is very rare.[1]

The clinical features of absent arterial pulsation in the upper limbs, head and neck, angiographic evidence of bilateral common carotid and subclavian arterial occlusion in our case are typical of aortic arch syndrome of Takayasu's arteritis. In our case, the occlusion of the inferotemporal retinal artery was noted clinically and confirmed by FFA. There was no evidence of emboli occlusion. The iris neovascularization was due to hypoxic retina. The visual acuity in both eyes and blood pressure in the arms had increased slightly with oral steroid therapy. The patient failed to turn up for follow-up after five months. So, the limitation of our study is that we could not evaluate the sequel of iris neovascularization.

Retinal artery occlusion in TA is very rare, as we have found only one previous report from north India in MEDLINE search. A high index of suspicion is therefore required to correlate the association of TA with branch retinal artery occlusion in a young patient.
